# Supplementary material for: Time-reversing a monochromatic subwavelength optical focus by optical phase conjugation of multiply-scattered light
Source: Sci Rep. 2017 Jan 30;7:41384. doi: 10.1038/srep41384 (PMC5278350; doi:10.1038/srep41384)
Supplement: Supplementary Information [file srep41384-s1.pdf]

Supplementary Information

Time-reversing a monochromatic subwavelength optical focus by optical phase conjugation of multiply-scattered light

Jongchan Park,[1] Chunghyun Park,[1,2] KyeoReh Lee, [1] Yong-Hoon Cho,[1,2,†] and YongKeun Park[1,*]

[1]Department of Physics, Korea Advanced Institute of Science and Technology, Daejeon 305-701, Republic of Korea.

[2]KAIST Institute for the NanoCentury, Korea Advanced Institute of Science and Technology, Daejeon 305–701, Republic of Korea.

Correspondence:

*Prof. YongKeun Park,

Department of Physics, Korea Advanced Institute of Science and Technology, Daejeon 305-701, Republic of Korea. Tel: (82) 42-350-2514, Email: yk.park@kaist.ac.kr

[†]Prof. Yong-Hoon Cho

Department of Physics, Korea Advanced Institute of Science and Technology, Daejeon 305-701, Republic of Korea. Tel: (82) 42-350-2549, Email: yhc@kaist.ac.kr

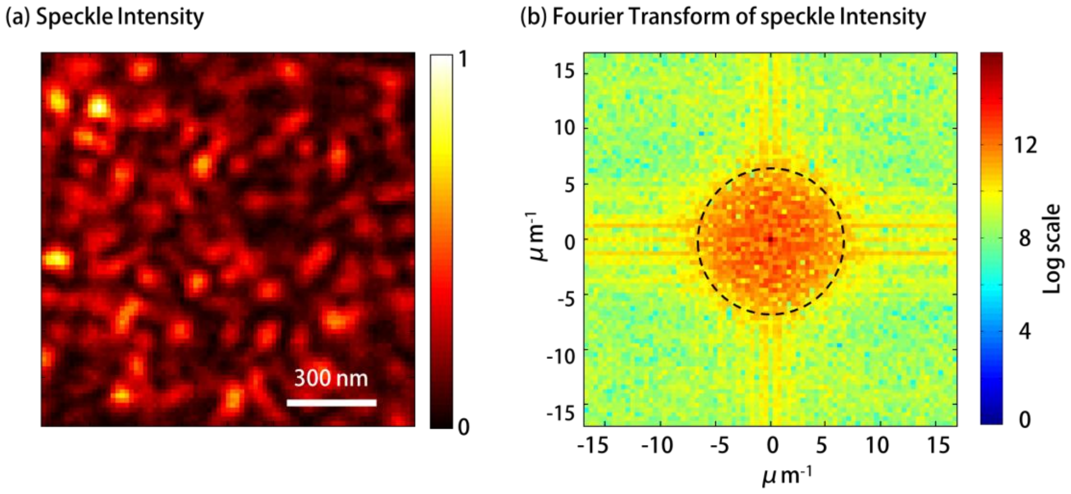

Supplementary Figure S1. Images of (a) speckle intensity and (b) Fourier Transform of speckle intensity. The dotted circle in (b) is corresponding to a spatial frequency of 1/150 nm.

Figure S1(a) shows the near-field speckle pattern generated from a disordered medium consists of zirconium dioxide random nanoparticles by illuminating it with a plane wave (λ = 532 nm). Figure S2(b) is the Fourier transformed image of fig. S1(a). The dotted circle on the Fourier Transformed image is corresponding to a spatial frequency of 1/150 nm. As shown in the figures, the addressable spatial frequency limit of our system is about 1/150 nm which is consistent with our experimental results where the size of the time-reversed focus was about 150 nm.

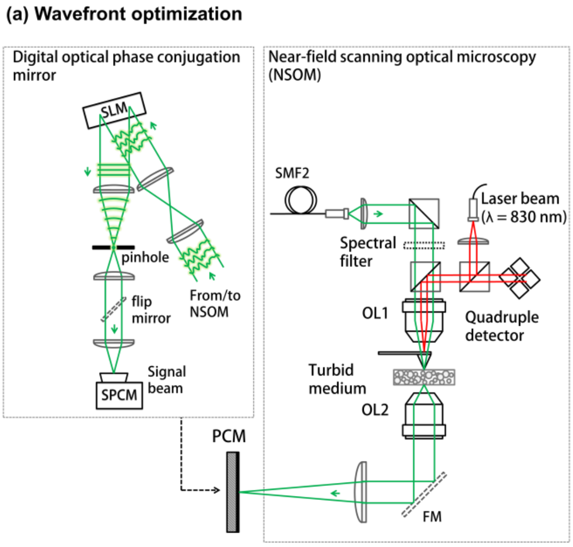

Supplementary Figure S2. Experimental scheme for (a) wavefront optimization process and (b) phase conjugation process. SMF: single mode fiber, SPCM: single photon counting module, P: polarizer, SLM: spatial light modulator, PMT: photomultiplier tube, FM: flip mirror, OL: objective lens, CCD: charge-coupled device.
